# Applications of 2-deoxy-2-fluoro-D-glucose (FDG) in Plant Imaging: Past, Present, and Future

**DOI:** 10.3389/fpls.2016.00483

**Published:** 2016-05-09

**Authors:** Amol Fatangare, Aleš Svatoš

**Affiliations:** Research Group Mass Spectrometry/Proteomics, Max Planck Institute for Chemical EcologyJena, Germany

**Keywords:** 2-deoxy-2-fluoro-D-glucose, FDG metabolism, F-sucrose, *in vivo* imaging, PET, photoassimilate translocation, carbon allocation

## Abstract

The aim of this review article is to explore and establish the current status of 2-deoxy-2-fluoro-D-glucose (FDG) applications in plant imaging. In the present article, we review the previous literature on its experimental merits to formulate a consistent and inclusive picture of FDG applications in plant-imaging research. 2-deoxy-2-fluoro-D-glucose is a [^18^F]fluorine-labeled glucose analog in which C-2 hydroxyl group has been replaced by a positron-emitting [^18^F] radioisotope. As FDG is a positron-emitting radiotracer, it could be used in *in vivo* imaging studies. FDG mimics glucose chemically and structurally. Its uptake and distribution are found to be similar to those of glucose in animal models. FDG is commonly used as a radiotracer for glucose in medical diagnostics and *in vivo* animal imaging studies but rarely in plant imaging. Tsuji et al. (2002) first reported FDG uptake and distribution in tomato plants. Later, Hattori et al. (2008) described FDG translocation in intact sorghum plants and suggested that it could be used as a tracer for photoassimilate translocation in plants. These findings raised interest among other plant scientists, which has resulted in a recent surge of articles involving the use of FDG as a tracer in plants. There have been seven studies describing FDG-imaging applications in plants. These studies describe FDG applications ranging from monitoring radiotracer translocation to analyzing solute transport, root uptake, photoassimilate tracing, carbon allocation, and glycoside biosynthesis. Fatangare et al. (2015) recently characterized FDG metabolism in plants; such knowledge is crucial to understanding and validating the application of FDG in plant imaging research. Recent FDG studies significantly advance our understanding of FDG translocation and metabolism in plants but also raise new questions. Here, we take a look at all the previous results to form a comprehensive picture of FDG translocation, metabolism, and applications in plants. In conclusion, we summarize current knowledge, discuss possible implications and limitations of previous studies, point to open questions in the field, and comment on the outlook for FDG applications in plant imaging.

## Introduction

The field of radiotracer imaging involves the application of radioisotope-labeled compound (radiotracer) to analyze the uptake and distribution of corresponding non-labeled analog, thus helping to shed light on the underlying physiology or diagnostics. It incorporates the application of both long-lived (e.g., ^14^C, ^32^P, ^35^S, etc.) and short-lived (e.g., ^11^C, ^13^N, ^15^O, ^18^F, etc.) radioisotope-labeled compounds. Many long-lived radioisotopes emit low-energy beta particles which cannot escape thick tissue. Thus, the destructive harvesting of tissue sample is necessary at the end of a labeling period for an analysis of radiotracer distribution (Webb and Gorham, 1964; Margolis et al., 1991; Finazzo et al., 1994). This limits the number of cases where long-lived radiotracers can be used to elucidate *in vivo* radiotracer distribution pattern (*in vivo* imaging). On the other hand, *in vivo* imaging could be easily achieved using positron-emitting short-lived radiotracers [positron emission tomography (PET)-radiotracers]. PET-radiotracer emits positron which, upon its impact with and consequential annihilation by an electron, give rise to two opposite anti-parallel high-energy gamma ray photons. The resulting gamma photons are able to penetrate thick tissue and allow for the spatiotemporal localization of radioisotopes, and thus of the corresponding PET-radiotracer, without destructive tissue sampling. Furthermore, due to the short-half lives of positron-emitting radioisotopes, ranging from minutes to several hours, radioactivity disappears quickly from the subject tissue. This rapid disappearance makes PET-radiotracers amenable to use in *in vivo* imaging studies without long-lasting radioactivity effects. One such PET-radiotracers, 2-deoxy-2-[^18^F]fluoro-D-glucose (^18^FDG, though for the current review, we simply refer it to as FDG) has been extensively used in *in vivo* clinical or animal studies to study tumor diagnostics, functional brain imaging, disease progression, and physiological and biochemical pathways (Alavi et al., 1982; Barrio et al., 1990; Phelps, 2004; Schieferstein and Ross, 2013). Recently, FDG has attracted the attention of plant scientists as it has been used as a tracer for *in vivo* imaging in plants (Hattori et al., 2008; Fatangare et al., 2014; Partelová et al., 2014; Meldau et al., 2015). FDG has been proposed as a radiotracer for photoassimilate in plant imaging studies (Hattori et al., 2008; Ferrieri et al., 2012; Fatangare et al., 2014). However, few questions about FDG applications in plant imaging remain unanswered. More experimental research is being conducted to establish FDG as a radiotracer in plants for photoassimilate translocation. In this article, we review the previous literature on its experimental merits to formulate a consistent picture of FDG applications in plants imaging. We discuss the possible implications and limitations of previous studies and comment on the potential applications of FDG in plant research.

### 2-deoxy-2-[^18^F]fluoro-D-glucose

**FIGURE 1:**
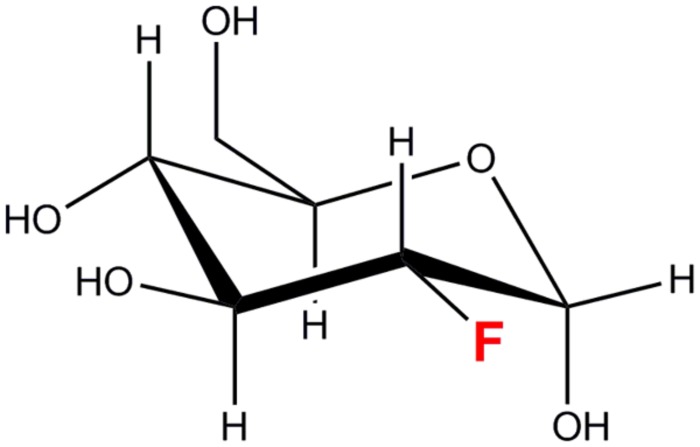
**2-deoxy-2-fluoro-D-glucose (^18^FDG/FDG)**.

2-deoxy-2-[^18^F]fluoro-D-glucose is a [^18^F]fluorine labeled glucose analog in which a hydroxyl group at the C-2 position has been replaced by the [^18^F] radioisotope (**Figure 1**). [^18^F] is a positron-emitting radioisotope with a half-life (*t*_1/2_) of 109.8 min. [^18^F] is synthesized in a cyclotron facility by a ^18^O(p,n)^18^F reaction (Ruth and Wolf, 1979) as depicted below.

O18+p1→F18+n1

After it is produced, [^18^F] is retained on a light quaternary ammonium anion exchange Sep-Pak column and can be eluted with an acetonitrile solution of Kryptofix[222]^TM^ and potassium carbonate. A nucleophilic substitution of mannose triflate by [^18^F] with Kryptofix[222]^TM^ as a catalyst yields FDG (Hamacher et al., 1986; Yu, 2006; Schubiger et al., 2007). Finally, FDG is purified from the reaction mixture using the series of following columns: an anion exchange column, a C-18 reverse phase column and an alumina column (Yu, 2006; Schubiger et al., 2007). Due to a short half-life of [^18^F] and the risk of radioactivity exposure, FDG synthesis is automated. Most automatic synthesizers can routinely produce FDG yields in the range of 55–60% within an hour from the starting of the synthesis (Gomzina et al., 2002; Yu, 2006; Schubiger et al., 2007). Once made, FDG is readily transported and utilized in clinical or lab facilities for PET studies. Owing to its longer half-life in comparison with other PET-radiotracers (for e.g.: ^11^C, *t*_1/2_ = 20.4 min; ^13^N, *t*_1/2_ = 9.96 min), FDG is a suitable radiotracer for *in vivo* imaging studies spanning several hours. In addition, the mean dispersion range of emitted positrons is shortest, thus allowing resolution in millimeter range (Sanchez-Crespo et al., 2004). FDG chemically and structurally mimics glucose and its uptake, and its distribution is found to be similar to that of glucose in animal models (Som et al., 1977, 1980). FDG is commonly used as a surrogate for radioactive glucose in medical diagnostics and animal studies to trace the uptake and metabolism of glucose in metabolically active tissue such as brain tissue or cancer cells (Som et al., 1980; Alavi et al., 1982; Ung et al., 2007).

### Past Research on FDG Applications in Plants

In total, there have been seven studies describing FDG applications in plants. Tsuji et al. (2002) first reported FDG uptake and distribution in tomato plants (Tsuji et al., 2002). Later, Hattori et al. (2008) described FDG translocation in intact sorghum plants and suggested that it could be used as a tracer for photoassimilate translocation in plants (Hattori et al., 2008). FDG has been used to study glycoside biosynthesis in plants as a measure of plant response to defense induction (Ferrieri et al., 2012). Recently, FDG has been utilized as a tracer for the root-mediated uptake of glucosamine (glucosamine is a major component of amino sugar nitrogen (ASN) base in the soil) (Li et al., 2014). FDG has been used to visualize and quantify the transport of solutes in plant tissue using PET imaging (Partelová et al., 2014). We have compared the radioactivity distribution of FDG to that of Ga-citrate and showed that FDG’s distribution pattern and translocation route is distinct from other radiotracers on account of its chemical specificity (Fatangare et al., 2014). Meldau et al. (2015) have demonstrated that FDG can be used to study carbon allocation in *Nicotiana* plants that are being attacked by herbivores (Meldau et al., 2015). In collaboration with Meldau et al. (2015), we showed that FDG can be metabolized into disaccharide compound *in planta*. Meldau’s study has expanded the scope of applications of FDG by elucidating *in vivo* carbon allocation in plants subjected to various biotic and abiotic stresses. FDG uptake and metabolism in plant cells is crucial to understanding and furthering FDG applications in plant imaging. We have characterized the metabolism of FDG in the model plant species *Arabidopsis thaliana*; we found that FDG is taken up by plant cells and metabolized to various metabolites such as FDG-6-phosphate (FDG-6-P), 2-deoxy-2-fluoro-maltose (F-maltose), 2-deoxy-2-fluoro-gluconic acid (F-gluconic acid) and uridine-diphosphate-FDG (UDP-FDG) (Fatangare et al., 2015). These studies have significantly advanced our understanding of FDG translocation and metabolism in plants but have also raised new questions. Here, we take a look at the previous results to form a comprehensive picture of the translocation and metabolism of FDG, and its applications in plants.

## FDG Uptake and Translocation in Plants

**FIGURE 2:**
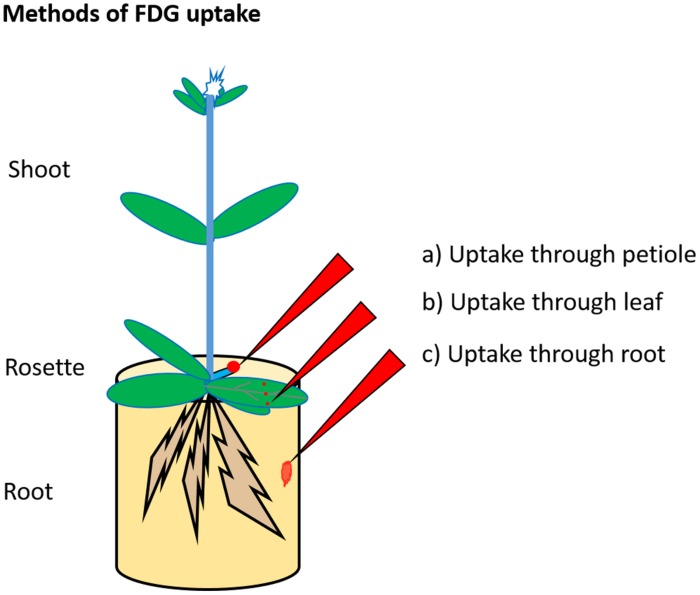
**Radiotracer uptake method.** FDG is supplied to the plant through cut petiole (a), pricked leaf (b), or root (c). In the first method, FDG solution is applied to the cut petiole. In the second method, the mature leaf is cut, pricked or scratched at the distal end, and FDG solution is applied to the wounded region. In the third method, FDG solution is added to an aqueous solution in which roots are placed.

Previous studies described three methods for promoting FDG uptake in the plant as depicted in **Figure 2.** The first “uptake through petiole,” involves cutting a mature leaf at a petiole and applying FDG solution to the cut end of the petiole toward the plant side (as in Tsuji et al., 2002; Ferrieri et al., 2012; Partelová et al., 2014). The second is “uptake through leaf”. This involves wounding (cut/scratch/prick) a mature leaf at the distal end and applying FDG solution to the wounded region (as in Hattori et al., 2008; Fatangare et al., 2014, 2015; Meldau et al., 2015). The third, “uptake through root,” involves dipping the plant’s root in FDG solution (as in Li et al., 2014). Upon uptake, the radioactivity associated with FDG translocates through the plant vasculature in the form of ‘FDG and/or its metabolites, which are cumulatively represented by [^18^F]-radioactivity. The resulting [^18^F]-radioactivity translocation pattern and translocation route depend upon the respective method of FDG uptake.

In the first method of FDG uptake (uptake through petiole), the percentage of [^18^F]-radioactivity translocated to other plant parts was significantly higher than that obtained by other methods. [^18^F]-radioactivity translocation occurred via the xylem (Tsuji et al., 2002). Cutting a leaf petiole results in tissue damage at the cut site. Such tissue damage normally leads to callous formation, which clogs and consequently blocks the transport of phloem. Xylem transport, however, remains relatively unaffected. Thus, the initial translocation of [^18^F]-radioactivity observed in such cases could be mainly ascribed to xylem transport. Radiotracer diffusion, xylem–phloem inter-transport and the possible involvement of phloem transport could be the factors shaping the observed distribution. Results obtained in Tsuji et al. (2002), Ferrieri et al. (2012), and Partelová et al. (2014) could be explained in light of the above assumption. In experiments by Partelová et al. (2014), a cut leaf petiole was dipped in FDG solution, and [^18^F]-radioactivity distribution in that leaf was monitored. [^18^F]-radioactivity was initially observed in leaf vascular tissue (major and minor leaf veins). In Partelová et al. (2014), the translocation must have occurred mainly via the xylem along with water transport under the influence of transpirational pull. The translocation of [^18^F]-radioactivity from the xylem to the leaf parenchyma tissue was low. The extent of [^18^F]-radioactivity translocation in the leaf parenchyma increased after high concentrations of glucose were added to the FDG solution used to feed the plants. Increased [^18^F]-radioactivity translocation can be attributed to the high osmotic pressure and ionic strength of the applied FDG and glucose mixture solution; these solutions resulted in a significant increase in FDG diffusion and its translocation in the leaf parenchyma (Partelová et al., 2014). In experiments by Ferrieri et al. (2012), a cut leaf petiole (on the side of a plant rosette) was dipped into FDG solution. In Ferrieri et al. (2012), [^18^F]-radioactivity translocated opposite to the direction of xylem transport, i.e., from the leaf to the plant stem. We think that in such a case, the initial [^18^F]-radioactivity translocation from petiole to stem may have occurred mainly through the xylem. Both the diffusion mechanism and the reverse pull on xylem flow in the petiole arising from the transpiration pull from other above-ground parts may contributed to this process. [^18^F]-radioactivity was also observed in root or parts which were situated below the fed petiole, an observation which could be only explained by [^18^F]-radioactivity translocation via the phloem. Imaging results showed that the [^18^F]-radioactivity translocated to both young and mature orthostichous leaves. These observations indicate the involvement of phloem transport in [^18^F]-radioactivity translocation. A fraction of [^18^F]-radioactivity must have been translocated via the phloem. FDG may enter the phloem at the cut petiole-end via direct loading or it may load along the petiole via xylem–phloem inter-transport. However, these assumptions should be experimentally tested. If true, the relative contribution of the xylem vs. phloem transport in net [^18^F]-radioactivity translocation is not clear and should be subject to further analysis.

In the second method of FDG uptake (uptake through leaf), a mature leaf was cut at the distal end, and the cut end was dipped into the FDG solution (as in Hattori et al., 2008). High [^18^F]-radioactivity was seen in the leaf lamina near the site of FDG application. [^18^F]-radioactivity in the other parts of the leaf blade was mainly confined to vascular tissue (major and minor leaf veins) and did not appear in the leaf lamina. Similar results were noted by Rotsch et al. (2015) using fluorine-labeled sucrose (F-sucrose). We think that localized FDG uptake resulted in observed high [^18^F]-radioactivity in the leaf lamina near the FDG application site and a fraction of the [^18^F]-radioactivity was loaded into the phloem mainly via the apoplastic-route of phloem loading. In Fatangare et al. (2014), experimenters pricked mature leaf on three spots: two spots on the leaf lamina and one spot on the leaf midrib. Here, FDG [^18^F]-radioactivity applied to leaf-lamina spots may distinctly translocate via the phloem, whereas the [^18^F]-radioactivity applied on the midrib may have a mixed translocation route (via both the phloem and xylem). [^18^F]-radioactivity preferentially translocated to young leaves, shoots, and roots. Stem-girdling experiments showed that the passage of [^18^F]-radioactivity was hindered at the girdling site. These observations suggest that the translocation of [^18^F]-radioactivity occurred exclusively via phloem. In Meldau et al. (2015), FDG was applied to two spots on the leaf lamina. This method avoided the direct introduction of radiotracer into xylem flow by excluding the middle spot on the midrib. Observations from the above three papers (Hattori et al., 2008; Fatangare et al., 2014; Meldau et al., 2015) support the idea that [^18^F]-radioactivity, when applied through the leaf lamina, is translocated to other plant parts mainly via the phloem in a manner similar to that of photoassimilate. However, a few contradictions still exist. Meldau et al. (2015) found that [^18^F]-radioactivity translocated to roots and accumulated significantly in the root tips. These results partially match with photoassimilate-partitioning results monitored using ^11^CO_2_ application (Ferrieri et al., 2013; Schmidt et al., 2015). Like [^11^C], [^18^F]-radioactivity was allocated to root tips. However, the relative percentage of [^18^F]-radioactivity translocated to roots upon herbivory increased in FDG experiments, whereas it decreased in ^11^CO_2_ experiments. The mismatch between the distribution of [^18^F]-radioactivity vs. [^11^C]-radioactivity in response to plant herbivory raises important doubts about whether FDG is a true radiotracer for photoassimilate or its translocation is similar to that of photoassimilate because it is translocated via phloem vasculature.

In the third method of FDG uptake (uptake through root), plant roots were placed in FDG solution (as in Li et al., 2014; Partelová et al., 2014). Radiograms showed that very little [^18^F]-radioactivity was translocated to parts above-ground. The relative percentage of [^18^F]-radioactivity accumulated in roots made up 60–99% of the total [^18^F]-radioactivity. Partelová et al. (2014) explained that the root system represents a specific site for FDG binding and is protected by a selective barrier preventing FDG transfer from the root parenchymal cell to the conductive tissue. Plant cells have been shown to take up FDG from external media (Fatangare et al., 2014). Localized uptake of FDG by the root cells, in combination with the apoplastic pool of FDG in root hairs, may result in high [^18^F]-radioactivity in root. The passive apoplastic flow of FDG toward plant stele will be hindered by the Casparian strip barrier at the root endodermis. This barrier could be crossed only via the symplastic route, which requires FDG to be taken up inside the cell. Plant cells can take up FDG from external medium and metabolize it into FDG-6-P (Fatangare et al., 2015). FDG and FDG-metabolites accumulate inside the cells, resulting in localized concentrations of [^18^F]-radioactivity. FDG-6-P transport across cells may be hindered at plasmodesmata, thus restricting the passage of [^18^F]-radioactivity further to vascular tissue. In our opinion, this mechanism results in the highly localized concentration of [^18^F]-radioactivity observed in roots. The Casparian strip, which limits the translocation of solutes across the root endodermis, will form the selective FDG barrier. Partelová et al. (2014) proposed that [^18^F]-radioactivity transport occurs via bidirectional phloem flow. However, once across the Casparian strip, [^18^F]-radioactivity may load into the xylem or phloem to be translocated to above-ground plant parts. For this reason, we could not disregard the possibility of xylem transport in such a case.

### What is Translocation Entity?

The chemical form in which [^18^F]-radioactivity translocates via the plant vasculature has not yet been established. Ferrieri et al. (2012) performed soluble sugar analysis from radio-labeled petioles and distant plant parts. Their findings suggest that [^18^F]-radioactivity translocates via the plant vasculature in the form of intact FDG molecule. We performed EDTA exudate analysis using mass spectrometry (MS) to determine whether fluorine-containing compounds could be found in the exudate. We found intact FDG in the phloem exudate. Partelová et al. (2014) also suggested that FDG is transported via the phloem. These findings contradict the idea that plants do not generally transport monosaccharides such as glucose in the phloem. Partelová et al. (2014) supported their claim by citing the findings of van Bel and Hess (2008), which showed that many members of families Ranunculaceae and Papaveraceae translocated more than 80% of carbohydrates in the form of hexoses. Liu et al. (2012) disputed the finding of van Bel and Hess (2008) by demonstrating that sucrose is the prominent translocation component in the phloem, rather than hexoses (Liu et al., 2012). The appearance of hexoses in the phloem sap collected by the EDTA exudation method was a procedural artifact (Liu et al., 2012). However, Liu et al. (2012) also states that plant vasculature is not incompatible with the long-distance transport of hexoses but, rather, that hexoses (that is, reducing sugars) are excluded from entry into the phloem by various physiological processes. Thus, hexoses are not transported out of leaves under normal circumstances and do not have access to the phloem solely for mechanistic reasons. We performed Benedict’s and Tollen’s reagent tests, which showed that FDG is a non-reducing sugar analog. Being non-reducing sugar, FDG will not pose any harm to the phloem tissue. If FDG get loaded into the phloem, it can translocate via plant vasculature without adversely affecting transportation in the phloem. However, this assumption raises further questions regarding the loading and unloading mechanisms of FDG into the xylem or phloem which should be addressed.

## FDG Metabolism in Plants

Understanding FDG metabolism in plant tissue is crucial for validating the role of FDG as radiotracer for plant imaging. Previous research literature has described FDG imaging in plants but has not provided a comprehensive picture of FDG metabolism in plant cells.

2-Deoxy-2-fluoro-D-glucose has been used in clinical and diagnostics studies relating to animals for decades. FDG uptake and metabolism has been extensively studied in animal cells (McSheehy et al., 2000; Kaarstad et al., 2002; Southworth et al., 2003). In animal tissue, FDG is taken up by the cells via glucose transporters (Higashi et al., 1998; Brown et al., 1999; Avril, 2004; Yen et al., 2004) and phosphorylated to FDG-6-P by the action of hexokinase or glucokinase (Sols and Crane, 1954; Bessell et al., 1972; Smith, 2001). It was assumed that FDG-6-P underwent no further metabolism and simply accumulated inside the cell (Bessell and Thomas, 1973; Miller and Kiney, 1981; Reivich et al., 1985; Suolinna et al., 1986).

Previously, FDG metabolism in plant cells was presumed to be similar to that in animal cells (Hattori et al., 2008). Ferrieri et al. (2012) reported that FDG was incorporated into anthocyanin glycoside biosynthesis and used it as a measure of plant defense induction. In their paper, Ferrieri et al. (2012) also raised the possibility that there is another F-metabolite whose identity has not yet been discovered. On the basis of *A. thaliana* cell suspension experiments, we proposed that FDG, as the glucose analog, is taken up into the cells via a low-affinity, facilitated-diffusion process mediated by a HgCl_2_-sensitive protein carrier at high external FDG concentrations (Fatangare et al., 2014). Upon uptake, FDG metabolizes to various compounds. We putatively identified the presence of four different fluorine-containing metabolites (F-metabolites), F-gluconic acid, FDG-6-P, F-maltose, and UDP-FDG, as FDG-derived metabolites on the basis of exact mono-isotopic mass and MS/MS fragmentation analysis (Fatangare et al., 2015). Two of the compounds (FDG-6-P and F-maltose) were confirmed by NMR and two others (F-gluconic acid and UDP-FDG) were putatively identified on the basis of high resolution MS data. Ferrieri et al. (2012) had proposed that FDG is incorporated into anthocyanin. However, we were not able to detect the ion for fluorine-containing anthocyanin glycosides (*m/z* 1344) in our negative or positive mode direct infusion MS or LC–MS data (Fatangare et al., 2015).

**FIGURE 3:**
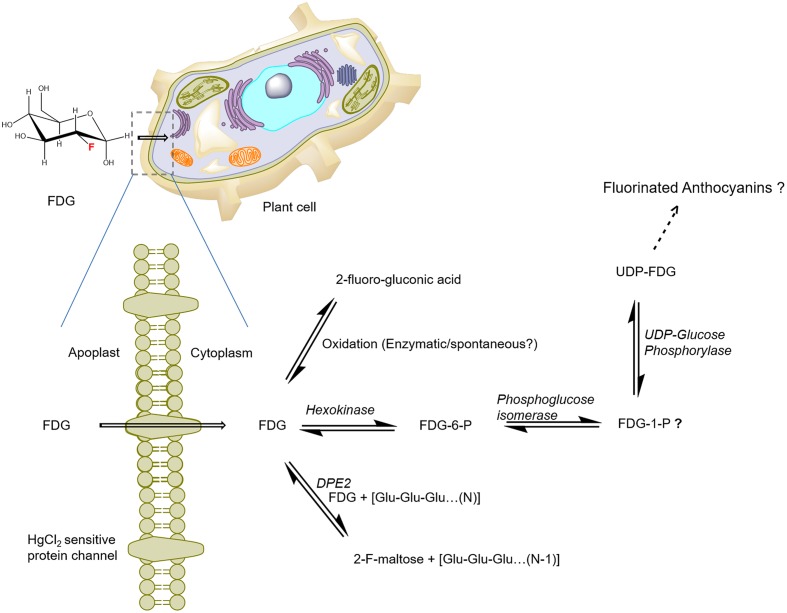
**Schematics of the potential route of FDG uptake and metabolism in plant cells.** FDG, 2-deoxy-2-fluoro-D-glucose; FDG-6-P, FDG-6-phosphate; FDG-1-P, FDG-1-phosphate; UDP, Uridine-diphosphate; F-maltose, 2-deoxy-2-fluoro-maltose; Glu, glucose; DPE2, *Arabidopsis* disproportionating enzyme 2 (modified from Fatangare et al., 2015).

As the current proposed model of FDG metabolism suggests (and as is depicted in **Figure 3**), upon uptake, FDG is transformed into FDG-6-P via hexokinase-mediated conversion; this process adds a negative charge to FDG and leads it to become trapped inside the cell. However, FDG is also transformed into various metabolites other than FDG-6-P. How F-gluconic acid is formed remains unknown. The biosynthesis of F-maltose seems to occur via the cytosolic component of the starch breakdown pathway (Fatangare et al., 2015). FDG could be converted into the F-maltose *in vitro* using the DPE2-mediated trans-glycosylation reaction with glycogen acting as a glucosyl donor (Tantanarat et al., 2012). The same DPE2-mediated trans-glycosylation reaction mechanism is likely involved in the biosynthesis of F-maltose. Previous studies have demonstrated nucleotide-bound forms of FDG (Schmidt et al., 1978; Kanazawa et al., 1996; Southworth et al., 2003) in animal tissues. We were able to show conclusively that UDP-FDG is a nucleotide-bound form of FDG. The biosynthesis of UDP-FDG in plants might be similar to that described by Kanazawa et al. (1997). UDP-glucose acts as a glucosyl moiety donor in various pathways such as those responsible for the biosynthesis of starch, anthocyanin or flavonoids. Considering the role of UDP-glucose in diverse pathways, we propose that UDP-FDG may have been involved in the biosynthesis of fluorinated anthocyanin (Ferrieri et al., 2012).

FDG-6-P is known to reversibly epimerize into 2-deoxy-2-fluoro-D-mannose-6-phosphate (FDM-6-P) by the catalytic action of phosphoglucose isomerase (Kanazawa et al., 1986; Kojima et al., 1988; Pouremad and Wyrwicz, 1991; O’Connell and London, 1995). 2-Deoxy-2-fluoro-D-mannose (FDM) metabolites such as FDM-1-P, FDM-1,6-biP and nucleotide diphosphate-FDM (NDP-FDM) have been reported in animal tissue (Kanazawa et al., 1996; Southworth et al., 2003). In their study, Fatangare et al. (2015) did not deny the presence of corresponding FDM-metabolites. Not all F-metabolites are identified due to their low abundance or limitations of MS and NMR detection techniques (Fatangare et al., 2015). Thus, there might be many more undiscovered end-products of FDG metabolism in plant cells.

To explain the observed FDG metabolism, we propose that upon intracellular uptake, FDG is considered as an energy source by the cell and is thus taken into the glycolytic pathway leading to the synthesis of FDG-6-P. However, all the FDG could not be metabolized into FDG-6-P, as building up levels of FDG-6-P inside the cell slows down the transformation of the compound through the feedback inhibition of hexokinase. FDG-6-P actually becomes a catabolic block that brings glycolysis to a halt. This process, which has already been shown in hypoxic animal tissue (Datema et al., 1980; Kurtoglu et al., 2007), may cause the rest of the free FDG to be pushed into F-maltose or F-gluconic acid biosynthetic pathways (**Figure 3**). FDG-6-P may be further transformed into FDG-1-P and finally to UDP-FDG as depicted in **Figure 3.** UDP-FDG may also be involved in the formation of fluorinated anthocyanins. The formation of various fluorine-metabolites in plants can be a way for plants to cope up with high intracellular concentrations of FDG, a known glycolytic inhibitor. Thus, the biosynthesis of various F-metabolites could be viewed as a way to use FDG as an energy source and/or a corrective–protective mechanism in the plant cells to counteract its consequences (Fatangare et al., 2015).

## Role of Pet in Plant Imaging

Positron-emitting short-lived radiotracers make *in vivo* imaging possible. In previous plant radiotracer imaging experiments, radiotracer localization was generally monitored using a photostimulable phosphor-coated imaging plate (IP) to obtain a static image of radiotracer distribution (Thorpe et al., 2007; Hattori et al., 2008; Ferrieri et al., 2012). Alternatively, the positron-emitting radiotracer imaging system (PETIS; Nakanishi et al., 1999; Mori et al., 2000; Tsuji et al., 2002; Matsuhashi et al., 2005), the planar positron imaging system (PPIS) (Uchida et al., 2004; Matsuhashi et al., 2006) or a PET scanner have been employed to study dynamic radiotracer distribution (Jahnke et al., 2009; Converse et al., 2013; De Schepper et al., 2013; Partelová et al., 2014). 2-Dimensional (2-D) imaging systems such as IP, PETIS, or PPIS are well-suited for planar structures such as plant leaves but cannot be employed for whole plants, which require 3-D imaging capability. This limitation can be overcome with the help of modern PET scanners. Unlike IP, PETIS, or PPIS, a PET scanner can capture images of 3-D radiotracer distribution over time *in vivo*. Radiotracer dynamics information obtained from PET scanner can be complemented with corresponding anatomical or morphological information to provide the spatial distribution of metabolic or biochemical activity which is precisely aligned with underlying tissue (Jahnke et al., 2009; Converse et al., 2013). Anatomical information can be acquired using techniques such as magnetic resonance imaging (MRI; Jahnke et al., 2009; Borisjuk et al., 2012) or X-ray-computed tomography (CT; Dhondt et al., 2010). Jahnke et al. (2009) demonstrated a MRI-PET co-registration system which combined the anatomical information of plants structures obtained from MRI with [^11^C]-radioactivity information obtained from PET. The drawback of the MRI–PET combination was that plants had to be measured separately in MRI and PET scanners and thus experienced different external environments during the experimental period. Converse et al. (2013) have used a microPET scanner to analyze the distribution of [^18^F]-radio-isotope in *Brassica oleracea*. However, their method lacked anatomical information from the corresponding plant. Partelová et al. (2014) have demonstrated the application of a microPET scanner to trace FDG in *in vivo* plant imaging. Fatangare et al. (2014) used a bi-functional PET/CT modality which coupled the morphological information of a plant derived from CT with the corresponding radioactive signal derived from PET to generate 4-D radiotracer dynamics. Integrated PET/CT modality is an effective way to address the above concerns. Because PET and CT scanners are embedded in the same instrument, the plant does not have to be transferred during PET/CT measurement and so the external environments of the plants remain the same. The fusion of the PET and CT images has been an effective way to deliver quantitative radioactivity information about various plant parts (Fatangare et al., 2014).

The radioactivity results obtained from PET/CT match the IP results. However, many limitations have also been noted in PET/CT imaging. The resolution provided by PET/CT is around 5- to 10-fold lower than the resolution provided by the IP imaging. This is evident when IP images are compared with PET/CT images. PET/CT images are not sharp and show a halo effect around the regions containing high radioactivity. Thin planar leaf morphology results in a high number of escaping positrons (Alexoff et al., 2011). Algorithms commonly used to calculate radioactivity are not yet optimized to account for these errors. So, new algorithms will be necessary: those that can correct the errors in radioactivity concentrations by integrating activity along the PET axis perpendicular to the leaf surface, including the detection of escaped positrons, and by calculating concentrations using a measured leaf thickness (Alexoff et al., 2011). Despite these limitations (low resolution, quantitative radioactivity calculation errors, and technical difficulties), the strength of PET/CT lies in its ability to elucidate *in vivo* radioactivity translocation. It is a powerful technique for deciphering the allocation of carbon in plants on a systemic (whole-plant) scale. The application of PET/CT will allow changing carbon dynamics or flux allocation to be monitored in the various parts of the plant as per the external environmental conditions or applied biotic and abiotic stresses as exemplified in Ferrieri et al. (2012) and Meldau et al. (2015).

## Current Status of FDG Applications in Plant Imaging

New radiotracers have tremendous impacts on medicine and biology. Thus, the development of new radiotracers and the discovery of novel applications of pre-existing radiotracers and improving radiotracer imaging techniques are major areas in current radiotracer research.

2-Deoxy-2-fluoro-D-glucose is already being used as a tracer in plant imaging research. It was employed as a tracer for photoassimilate (Hattori et al., 2008), or glycoside biosynthesis, or (Ferrieri et al., 2012), or root-mediated uptake of glucosamine (Li et al., 2014), or the visualization and quantification of solute transport in plant tissue (Partelová et al., 2014), or studying carbon allocation (Meldau et al., 2015). This great diversity of FDG applications arises from the different methods of FDG uptake and the purpose experimenters have in mind. There are two main lines of thought concerning these methods: first, when FDG is supplied through roots, it behaves as an entity dissolved in water and it mimics the xylem-mediated transport of solutes. This method could be used to measure glucose uptake by roots and its translocation rate via the xylem. Casparian strip; however, will impede FDG translocation and result in highly localized concentrations of FDG in roots compared to neighboring parts. Thus, absolute quantification in those neighboring parts will be difficult. Second, when FDG is supplied through leaves, it is taken up and metabolized like glucose in the leaf cells, giving rise to a multitude of fluorinated metabolites. However, in both of the above scenarios, a fraction of FDG also translocates via plant vasculature. Meldau et al. (2015) found that [^18^F]-radioactivity allocation in *Nicotiana attenuata* root tips decreased after simulated herbivory. This result matches with the Schmidt et al.’s (2015) finding that recently fixed [^11^C] carbon allocation in secondary roots tips decreased after herbivory. The net [^11^C] carbon allocation to roots, as opposed to [^18^F]-radioactivity, was decreased after herbivory (Schmidt et al., 2015). In our opinion, FDG behaves as an entity that has been translocated via the phloem giving rise to translocation patterns that are similar to but not exactly the same as patterns of photoassimilate. Comparative imaging between ^18^FDG and ^11^CO_2_ may be the best way to check whether [^18^F]-radioactivity allocation can represent [^11^C] photoassimilate allocation. Translocation patterns, however, differ greatly from plant to plant. Thus, we propose to employ a single plant to conduct such comparative imaging experiments using PET/CT. Because PET/CT allows for non-invasive radiotracer monitoring, a single plant could be used in two successive labeling experiments, first by ^11^CO_2_ and second by FDG to compare the resultant radioactivity translocations (Fatangare et al., 2014).

## Questions and Considerations in Future FDG Experiments

Optimizing the method of radiotracer introduction is one of the important considerations for future experiments. If FDG can be applied to leaf lamina without damaging the vascular tissue, its translocation will be confined to the phloem. The resulting radiotracer translocation will be representative of phloem translocation. For leaf uptake experiments, the best way to apply FDG is to gently prick the leaf on the abaxial leaf lamina. Pricked spots should be distant from the midrib and should cause minimal damage to nearby minor or major leaf veins. Isotonic FDG solution (2–5 μL, ∼1–5 MBq/μl) should be applied at the sites. The removal of the cuticle using abrasive paste or sandpaper could be another way to improve the FDG uptake in leaf tissue. For root uptake experiments, FDG solution (20–100 MBq in total) should be applied to roots. Prior to imaging, FDG solution should be removed; roots should be washed in distilled water to remove unbound FDG and then placed in non-radioactive nutrient medium. If compatible with the experimental aim, one may remove the FDG applied leaf or root prior to imaging the plants. This will greatly reduce the halo effect produced by high [^18^F]-radioactivity localized the leaf or root to which FDG has been applied. Plant stage is one of the important determinants influencing carbon allocation patterns. Thus for all comparative experiments it is important to use individuals from one plant species at the same growth stage.

There are still a few unanswered questions in this field. First of all, it is known that FDG causes cytotoxicity in hypoxic tumor cells (Lampidis et al., 2006; Kurtoglu et al., 2007). In leaf uptake experiments, the localized application of FDG may result in high cytoplasmic concentration of FDG in the surrounding plant cells which in turn lead to cytotoxic effects (Fatangare et al., 2015). Thus, the level at which FDG becomes toxic to for plant cells should be investigated. After FDG application, [^18^F]-radioactivity spreads through the leaf lamina. The relative contribution of symplastic and apoplastic transport routes in [^18^F]-radioactivity spread in the leaf lamina should be analyzed. FDG is taken up by plant cells and metabolized to various metabolites. Although we have some idea of the metabolites and pathways involved, the picture of metabolism of FDG in plant cells is not yet complete. There might be many unknown FDG-derived metabolites that have not yet been reported. After uptake, [^18^F]-radioactivity is seen translocated to various plant parts via plant vasculature. Previous studies suggest that intact FDG may be a translocation entity. This conclusion is contrary to the idea that monosaccharides are generally not translocated via the phloem. Analysis of phloem exudate collected using aphid stylets will be good way to firmly establish the above claim. Furthermore, one important question remains: how FDG, despite being a monosaccharide glucose analog, is loaded/unloaded in the plant vascular system. This question need to be addressed.

Apart from FDG, various other F-sugars have also been utilized in plant research. Being sugar analogs, F-sugars are recognized by various enzymes and transporters. Tantanarat et al. (2012) tested 1-deoxy-1-fluoroglucose, FDG, 3-deoxy-3-fluoroglucose (3-F-glucose), 4-deoxy-4-fluoroglucose, and 6-deoxy-6-fluoroglucose (6-F-glucose) as substrates for the DPE2 enzyme from *A. thaliana* (AtDPE2). AtDPE2 was shown to transglycosylate FDG, 3-F-glucose, and 6-F-glucose to form corresponding F-disaccharide products (Tantanarat et al., 2012). 1-deoxy-1-flourofructose has been used as substrate for sucrose synthase (Card and Hitz, 1984; Römer et al., 2001). 6-deoxy-6-flourogalactose has been used to study the carbohydrate-binding property of lectin, a compound found in elderberry bark (Shibuya et al., 1987). 1-deoxy-1-fluorosucrose (1-F-sucrose), a poor substrate for invertase (Hitz et al., 1985), has been used in biochemical studies involving sucrose transport and metabolism in maize (Schmalstig and Hitz, 1987a,b; Felker and Goodwin, 1988). 1-F-sucrose is found to be a good substrate for transporters and sucrose synthase (Hitz et al., 1985, 1986). Thus, it has been used to analyze the substrate specificity of the enzymes that handle sucrose and transporters in the plant (Hitz et al., 1985, 1986; Damon et al., 1988; Lemoine et al., 1988; Römer et al., 2001). The synthesis of 6-deoxy-6-fluorosucrose (6-F-sucrose) was reported by Eklund and Robyt (Eklund and Robyt, 1988). Recently, a new route to 6-F-sucrose synthesis has been developed by Gao et al. (2014). Authors also proposed its application in plant imaging using PET (Gao et al., 2014). Rotsch et al. (2015) subsequently demonstrated that 6-F-sucrose translocation closely resembles ^14^C-sucrose translocation in maize leaves. We think 1-F-sucrose or 6-F-sucrose can be a useful alternative to trace the translocation of photoassimilate in plants and should be experimentally validated.

## Scope of FDG Applications

Understanding carbon allocation *in vivo* in crop plant is an important area of research relevant to the application of FDG in plant research. Carbon allocation dynamics in plants depends on the relative strengths of various sinks and sources. In the absence of any external influences, plants can be viewed as intricate systems of carbon sources and sinks. The complex interplay between various sinks and sources determines plant productivity in terms of biomass accumulated in harvested parts. However, the strength of various sinks and sources changes rapidly when plants are subjected to external biotic and abiotic factors. Such manipulation of carbon allocation dynamics in plants could be employed to improve crop productivity via controlled the alteration of photoassimilate partitioning. Understanding carbon allocation and associated flux changes *in vivo* in crop plant is key step in improving crops. FDG has already been used to trace the changes in carbon allocation, both on temporal and spatial scales. It can also be employed to study source–sink transition and to measure source–sink strengths. Because FDG and F-sucrose are fed in their intact form, their translocation does not involve the lag time required for carbon fixation as observed in ^11^CO_2_ experiments, and they can also be used to unravel carbon allocation in the dark phase when no photosynthesis can occur. FDG and/or its metabolites translocate via the phloem, so real-time monitoring of [^18^F]-radioactivity translocation may be used to measure rates of phloem transport. It may also be useful for deciphering vascular connectivity in a plant’s architecture. PET/CT easily allows for *in vivo* imaging in plants. We believe that FDG application in combination with PET/CT imaging in plants will play a key role in deciphering dynamic changes in carbon allocation pattern and fluxes in plants challenged by external biotic and abiotic stresses.

## Author Contributions

AF came up with idea and made rough manuscript draft. AS made the suggestions, corresponding changes, text editing and final corrections prior submission.

## Conflict of Interest Statement

The authors declare that the research was conducted in the absence of any commercial or financial relationships that could be construed as a potential conflict of interest.
